# Levels of sP-selectin and hs-CRP Decrease with Dietary Intervention with Selenium and Coenzyme Q10 Combined: A Secondary Analysis of a Randomized Clinical Trial

**DOI:** 10.1371/journal.pone.0137680

**Published:** 2015-09-16

**Authors:** Urban Alehagen, Tomas L. Lindahl, Jan Aaseth, Erland Svensson, Peter Johansson

**Affiliations:** 1 Department of Cardiology and, Department of Medical and Health Sciences, Linköping University, Linköping, Sweden; 2 Dept. of Clinical and Experimental Medicine, Linköping University, Linköping, Sweden; 3 Dept. of Clinical Chemistry, County Council of Östergötlan, Linköping, Sweden; 4 Research Department, Innlandet Hospital Trust and Hedmark University College, Tromsø, Norway; 5 Formerly Swedish Defence Research Agency, Stockholm, Sweden; Indiana University Richard M. Fairbanks School of Public Health, UNITED STATES

## Abstract

**Background/Objectives:**

Inflammation and oxidative stress are central in many disease states. The major anti-oxidative enzymes contain selenium. The selenium intake in Europe is low, and supplementation with selenium and coenzyme Q_10_, important anti-oxidants, was evaluated in a previous study. The aim of this study was to evaluate response on the inflammatory biomarkers C-reactive protein, and sP-selectin, and their possible impact on cardiovascular mortality.

**Subjects/Methods:**

437 elderly individuals were included in the study. Clinical examination, echocardiography, electrocardiography and blood samples were drawn. The intervention time was 48 months, and median follow-up was 5.2 years. The effects on inflammation/atherosclerosis were evaluated through analyses of CRP and sP-selectin. Evaluations of the effect of the intervention was performed using repeated measures of variance. All mortality was registered, and endpoints of mortality were assessed by Kaplan-Meier plots.

**Results:**

The placebo group showed a CRP level of 4.8 ng/mL at the start, and 5.1 ng/mL at the study end. The active supplementation group showed a CRP level of 4.1 ng/mL at the start, and 2.1 ng/mL at the study end. SP-selectin exhibited a level of 56.6 mg/mL at the start in the placebo group and 72.3 mg/mL at the study end, and in the active group the corresponding figures were 55.9 mg/mL and 58.0 mg/mL. A significantly smaller increase was demonstrated through repeated measurements of the two biomarkers in those on active supplementation. Active supplementation showed an effect on the CRP and sP-selectin levels, irrespective of the biomarker levels. Reduced cardiovascular mortality was demonstrated in both those with high and low levels of CRP and sP-selectin in the active supplementation group.

**Conclusion:**

CRP and sP-selectin showed significant changes reflecting effects on inflammation and atherosclerosis in those given selenium and coenzyme Q_10_ combined. A reduced cardiovascular mortality could be demonstrated in the active group, irrespective of biomarker level. This result should be regarded as hypothesis-generating, and it is hoped it will stimulate more research in the area.

## Introduction

Inflammation is part of the atherosclerotic process in the body[1], but also in other processes, including different disease states such as e.g. cardiometabolic syndrome, acute coronary syndromes [2, 3], heart failure with preserved ejection fraction [4, 5] as well as heart failure with reduced ejection fraction [6, 7] and also in aortic stenosis [8] and rheumatoid inflammations that may predispose a patient to cardiovascular disease. Aging itself is also associated with an increased inflammatory response [9]. An increased inflammatory response in disease states or in the elderly can be a marker of disease progression as well as an indicator of a poorer prognosis [10]

Inflammation and atherosclerosis are interrelated conditions that are also associated with increased oxidative stress in the body [11]. The selenium-containing enzymes glutathione peroxidase, selenoprotein P and thioredoxin reductase are some of the major contenders in the defence against oxidative stress in the body [12]. An association between ischemic heart disease and intake of selenium has been discussed for a long time, [13, 14]. However, there are also conflicting reports on the effectiveness of intervention with selenium in cardiovascular disease [15–19].

The intake of selenium is generally low in Europe, and has been estimated to be around 40 μg/day [20]. The required intake of selenium in order to obtain optimal function of the intracellular enzyme glutathione peroxidase, and the extracellular protector selenoprotein P has been discussed, and Xia et al. reported a needed intake of 75 μg/day of selenium for adult Caucasians [21]. During conditions of increased oxidative stress and inflammation there is an increased need for intake of selenium [22]. A possible association between cardiovascular disease and death and intake of selenium has been discussed in the literature. Salonen et al. reported a 2.9 fold increase in cardiovascular mortality in those with a low selenium intake [14]. However, conflicting reports regarding the effectiveness of intervention with selenium on cardiovascular disease have also been published [15–19].

Coenzyme Q_10_ (ubiquinone) is present in all living cells in the body where it is an important antioxidant, but is also active in the mitochondrial respiratory chain. However, the endogenous production of coenzyme Q_10_ declines after the age of 20, and in the myocardial cells it is reduced to about half at the age of 80 years [23]. As observed by Xia et al. there is an important interrelationship between selenium and coenzyme Q_10_, as selenium (selenocystein) is needed for the reduction of ubiquinone to ubiquinol, the active form of coenzyme Q_10_ [24, 25]. This interrelationship might explain the conflicting results in studies performed in areas with low selenium intake, where just one of the substances has been supplemented.

There is sparse information of the supplementation of selenium and coenzyme Q_10_ combined. However, Kuklinski, in a small study, reported lower mortality, a decreased plasma level of the biomarker N-terminal fragment of proBrain Natriuretic Peptide (NT-proBNP), and increased cardiac function according to echocardiography in patients with myocardial infarction who received supplementation with selenium and coenzyme Q_10_ in combination. We have recently presented data from a prospective double-blind, placebo controlled study of elderly Swedish healthy individuals, given dietary supplementation of either selenium and coenzyme Q_10_ or placebo during four years of intervention [26]. The result was a reduction of cardiovascular mortality, increased cardiovascular function as seen in echocardiography, and a smaller increase in NT-proBNP as a result of the combined intervention.

The rationale for the present analysis is that selenium and coenzyme Q_10_ are two major contenders in the human oxidative defence system [12, 27–29]; thus, a possible contributable mechanism to the positive results might be a decreased inflammatory response by the dietary supplementation of selenium and coenzyme Q_10_.

One of the most commonly used biomarkers for inflammatory response in clinical practice is C-reactive protein (CRP). CRP is an acute phase reactant and a general marker of systemic inflammation, but also a well-known biomarker for cardiovascular risk[30]. sP-selectin (CD62P) is a cell adhesion molecule expressed on activated endothelial cells and involved in their leukocyte attachment during inflammation [31]. The predominant part of sP-selectin found in plasma originates from activated platelets, and is a biomarker for atherosclerosis. sP-selectin has also been reported to give reliable prognostic information regarding the atherothrombotic process and cardiovascular risk [32, 33].

The aim of the present study was to evaluate the effect of selenium and coenzyme Q_10_ on the inflammatory response as reflected by CRP and sP-selectin. A second aim was to determine whether selenium and coenzyme Q_10_ had an impact on the association between inflammation and mortality.

## Methods

### Study population

This is a secondary analysis of a prospective randomized double-blind placebo-controlled trial in an elderly community population of 443 individuals with an age between 70–88 years that has been previously reported [26, 34]. The present evaluation of sP-selectin and CRP, is a post hoc analysis, whereas the evaluation the effect of the intervention on inflammation was prespecified.

The first participant was included in January 2003, and the last participant concluded the study in February 2010. All participants received the intervention for 48 months during which they were re-examined every six months. During the study, all mortality was registered. In the study, 221 individuals received active supplementation of 200 μg/day organic selenium (SelenoPrecise®, Pharma Nord, Denmark), plus 200 mg/day of coenzyme Q_10 (_Bio-Quinon®, Pharma Nord, Denmark), and 222 individuals received a placebo. At inclusion all participants went through a clinical examination, new patient records were obtained, the New York Heart Association functional class was assessed, and an ECG and Doppler-echocardiography was performed. Informed consent was obtained from each patient. The study was approved by the Regional Ethical Committee and conforms to the ethical guidelines of the 1975 Declaration of Helsinki. (The Medical Product Agency declined to review the study protocol since the study was not considered a trial of a medication for a certain disease but rather one of food supplement commodities that are commercially available). This study was registered at Clinicaltrials.gov, and has the identifier NCT01443780.

### Blood samples

Blood samples were collected while the participants were resting in supine position. Pre-chilled, EDTA-vials were used. The vials were centrifuged at 3000*g*, +4°C, and were then frozen at -70°C. No sample was thawed more than once.

### sP-selectin

Soluble sP-selectin (sCD62P) was analysed utilizing an ELISA from R&D (Abingdon, UK). The intra-assay coefficient of variation (CV) was about 5% and the inter-assay CV about 9%.

### C-reactive protein

hs-CRP was analysed on an Advia 1800 instrument (Siemens Healthcare Diagnostics, Stockholm, Sweden) using a latex-enhanced immunochemistry method with reagents from the same company. The detection limit was 0.12 mg/L. The total CV was 1.1% at 13.3 mg/L and 2.9% at 0.99 mg/L.

## Statistics

Descriptive data are presented as percentages or mean ± SD. The Student’s unpaired two-sided *T*-test was used for continuous variables. Kaplan-Meier analyses and plots of cardiovascular mortality for the period of up to 5.2 years were made separately for CRP and sP-selectin, each divided in two at their median levels. Censored participants were those still living at the end of the study, or who had died for reasons other than cardiovascular disease. Completed participants were those who had died due to cardiovascular disease. Evaluating the *P*-values between the two groups of mortality at 1900 days was based on lifetable analyses using cumulative proportional surviving, and standard error of cumulated survivals to obtain a z-value. Evaluation of the effects of treatment were based on the group mean, but where the values of the individual participant were identified during the three different measured time points using a repeated measures of variance analysis. *P*-values < 0.05 were considered significant, based on a two-sided evaluation. All data were analysed using standard software (Statistica v. 12.0, Statsoft Inc, Tulsa, OK, USA.).

## Results

**Fig 1:**
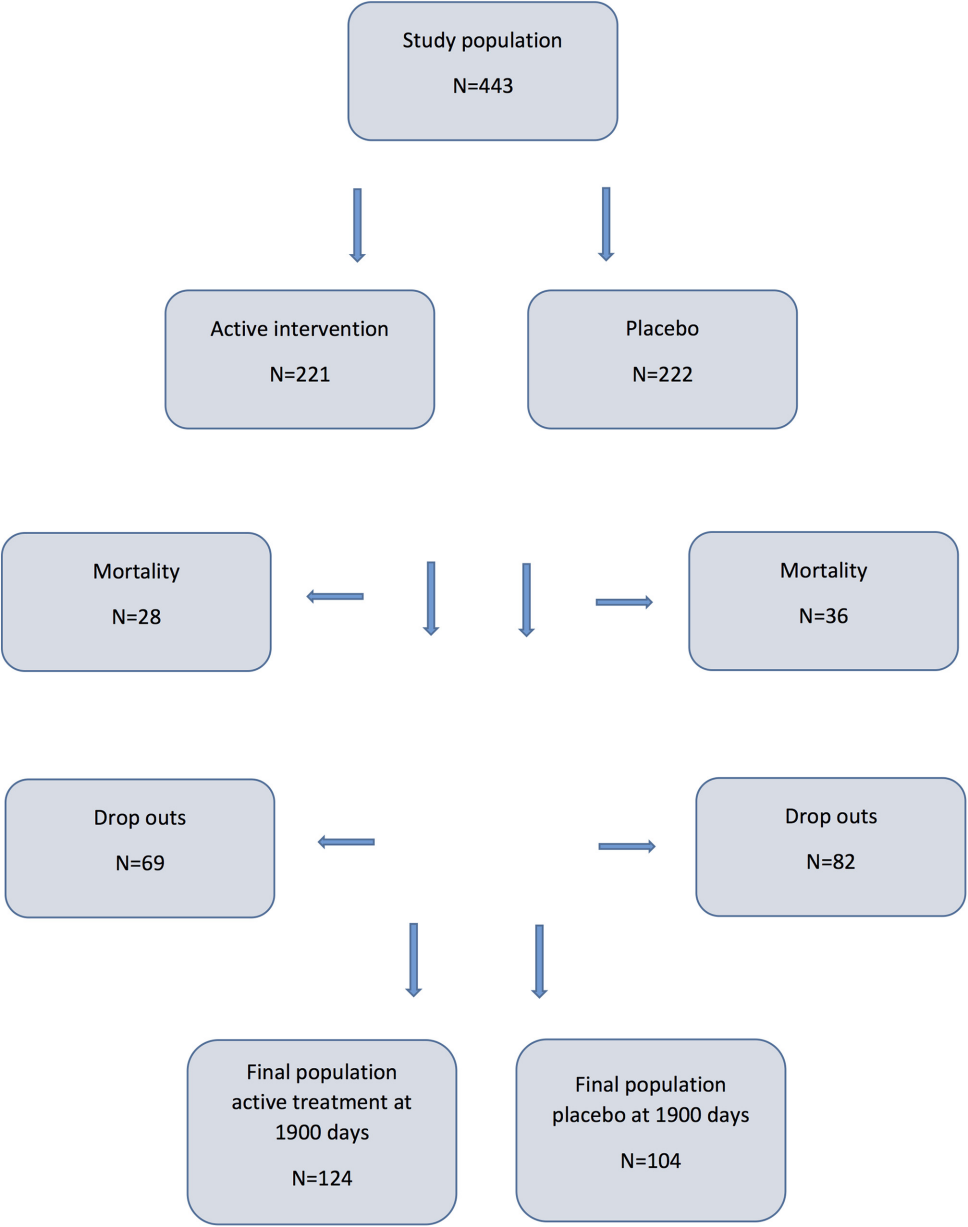
CONSORT diagram illustrating the design of the study.

The baseline characteristics of the study population are presented in Table 1, and a CONSORT flow chart of the study is presented in Fig 1.

**Table 1:** Baseline characteristics of the study population receiving intervention of a dietary supplementation of selenium and coenzyme Q10 combined during 4 years.

	Active	Placebo	p-value
N	216	221	
Age years mean (SD)	76.9 (3.5)	77.3 (3.4)	0.35
Males/Females n	112/104	110/111	
**History**			
Smokers (present) n (%)	20 (9.3)	20 (9.0)	0.94
Diabetes n (%)	46 (21.3)	48 (21.7)	0.91
Hypertension n (%)	155 (71.8)	168 (76.0)	0.31
IHD n (%)	45 (20.8)	52 (23.5)	0.50
NYHA class I n (%)	117 (54.2)	107 (48.4)	0.23
NYHA class II n (%)	58 (26.9)	64 (29.0)	0.62
NYHA class III n (%)	40 (18.5)	47 (21.3)	0.47
NYHA class IV n (%)	0	0	
**Medications**			
ACEI n (%)	32 (14.8)	53 (24.0)	0.02
ARB n (%)	10 (4.6)	13 (5.9)	0.56
Beta blockers n (%)	75 (34.7)	72 (32.6)	0.64
Digitalis n (%)	10 (4.6)	11 (5.0)	0.87
Diuretics n (%)	68 (31.5)	88 (39.8)	0.07
Statins n (%)	42 (19.4)	50 (22.6)	0.41
**Examinations**			
EF<40% n (%)	14 (6.5)	17 (7.7)	0.65
NT-proBNP ng/L mean (IQR)	537 (398)	516 (330)	0.86
sP-Selectin mg/mL mean (IQR)	55.9 (27.7)	56.6 (31.4)	0.75
CRP mg/mL mean (IQR)	4.0 (3.6)	4.9 (4.0)	0.45

Note: ACEI: ACE- inhibitors; ARB; Angiotension receptor blockers; EF: Ejection fraction; IHD; Ischemic heart disease; IQR: Inter quartile range; NT-proBNP: N-terminal fragment of proBNP; NYHA: New York Heart Association functional class; SD: Standard Deviation.

In the population, 437 individuals were evaluated as 6 participants of the 443 did not deliver blood samples for analyses of sP-selectin. Almost equal numbers of males versus females were found (222 males versus 215 females). The two groups (active treatment versus placebo) were well balanced at the start except regarding treatment with ACE-inhibitors, regarding which a significantly greater part were on treatment in the placebo group (14.8% versus 24.0%; *P* = 0.02).

In the placebo group the mean concentration of CRP at study start was 4.8 ng/mL, whereas the mean concentration at study end was 5.1 ng/mL (*P* = 0.91) (Table 2).

**Table 2:** Levels of C-reactive protein and sP-selectin at study start, after 18 months and at study end in the placebo group, and in the active treatment group.

	CRP baseline (mg/mL)	CRP 18 months (mg/mL)	CRP 48 months (mg/mL9	sP-selectin baseline (ng/ml)	sP-selectin 18 months (ng/mL)	sP-selectin 48 months (ng/mL)
**Placebo group, mean (SD)**	4.84 (12.0)	3.69 (3.7)	5.05 (6.2)	56.6 (26.3	57.9 (24.8)	72.3 (35.9)
**Active treatment, mean (SD)**	4.06 (11.7)	2.61 (3.2)	2.07 (2.3)	55.9 (22.4)	54.5 (21.1)	58.0 (19.2)

Note: CRP: C-reactive protein.

However, in the active treatment group the mean concentration of CRP at study start was 4.1 ng/mL and the concentration at study end was 2.1 ng/mL (t = -1.75; *P* = 0.08). Evaluating the soluble part of sP-selectin showed a concentration in the placebo group of 56.6 mg/mL at study start and a concentration of 72.3 mg/mL at study end (t = 4.39; *P*<0.0001). In the active treatment group a concentration of sP-selectin at study start of 55.9 mg/mL was found, and a concentration of 58.0 mg/mL at study end (t = 0.03; *P* = 0.97). Thus, a significant difference in sP-selectin concentration between the two groups during the intervention of 48 months could be seen, and a trend against difference comparing the concentration of CRP between the two groups.

**Fig 2:**
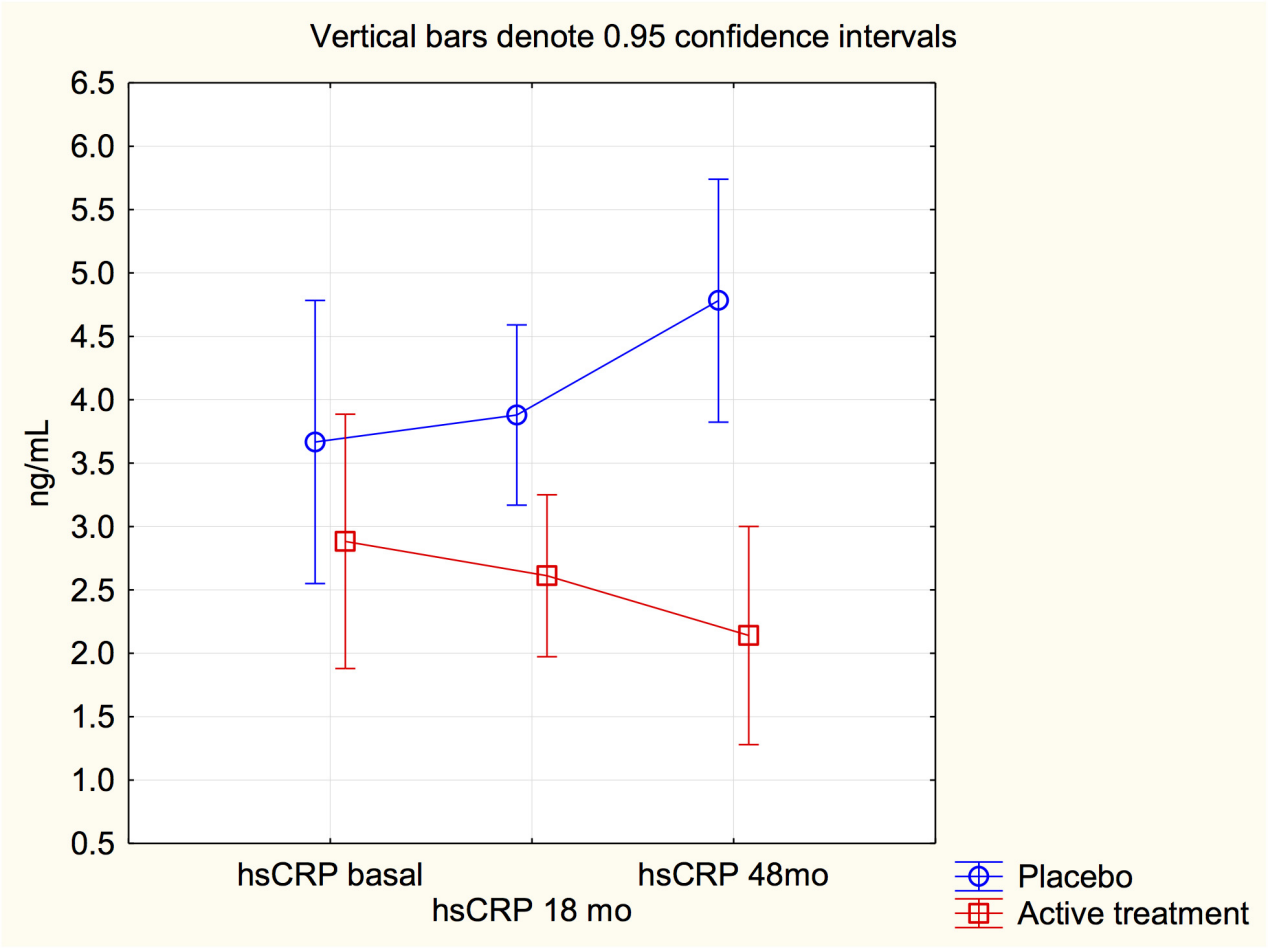
Graph illustrating C-reactive protein measurements at study start, after 18 months and at study stop at 48 months in the study population given selenium and coenzyme Q10 combined, or placebo. Note: Analysis according repeated measures of variance.

However, to evaluate a possible treatment effect a repeated measures of variance was performed. The model which included the two groups (active and placebo) and the measured levels at three different time points (baseline, 18 and 48 months) showed a significant treatment effect on the CRP level (F = 8.67; *P* = 0.004), indicating that a significant difference between active intervention and placebo could be found. Evaluation of the interaction revealed a significant interaction (F = 4.73; *P* = 0.009) indicating that the obtained treatment effect was not based on difference in CRP level at start, but to a significantly reduced level of CRP due to the intervention (Fig 2).

**Fig 3:**
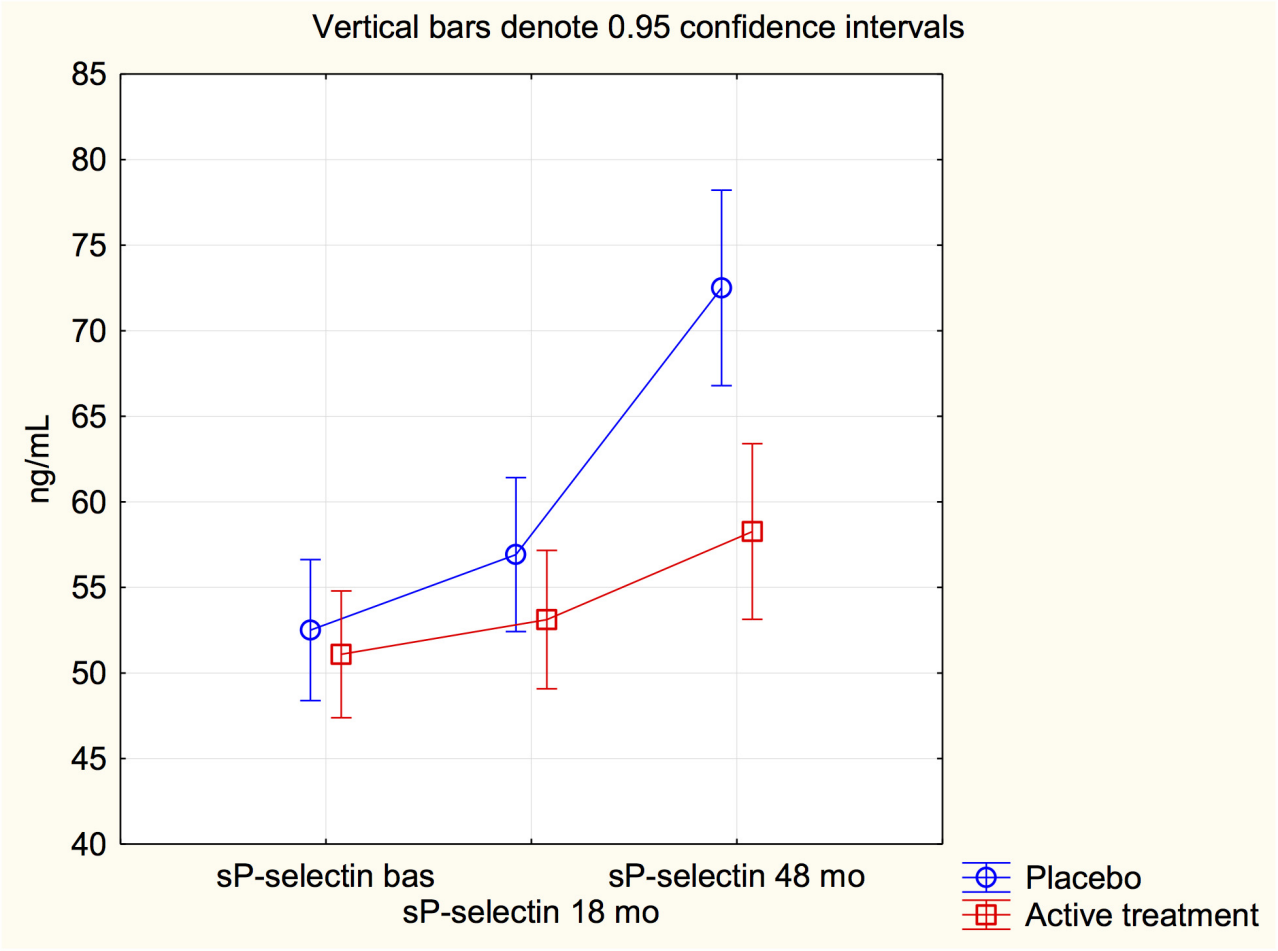
Graph illustrating sP-selectin measurements at study start, after 18 months and at study stop at 48 months in the study population given selenium and coenzyme Q10 combined, or placebo. Note: Analysis according repeated measures of variance.

Performing the same procedure on the sP-selectin level showed a significant treatment effect (F = 6.52; *P* = 0.01), and a significant interaction (F = 7.08; *P* = 0.0009). Thus a significant treatment effect as seen on the sP-selectin level (Fig 3).

### Association between inflammation and cardiovascular mortality

Of those having a CRP or sP-selectin concentration above or below the median value the distribution of cardiovascular death was examined as shown in Table 3.

**Table 3:** Distribution of cardiovascular mortality in placebo and active treatment groups above and below median value of C-reactive protein, and sP-selectin in the study population during 5 years of follow-up.

	CRP > median value	CRP < median value	sP-selectin > median value	sP-selectin < median value
**Active treatment**	8/103	5/113	8/104	5/112
**Placebo**	15/108	13/113	16/111	12/110

**Fig 4:**
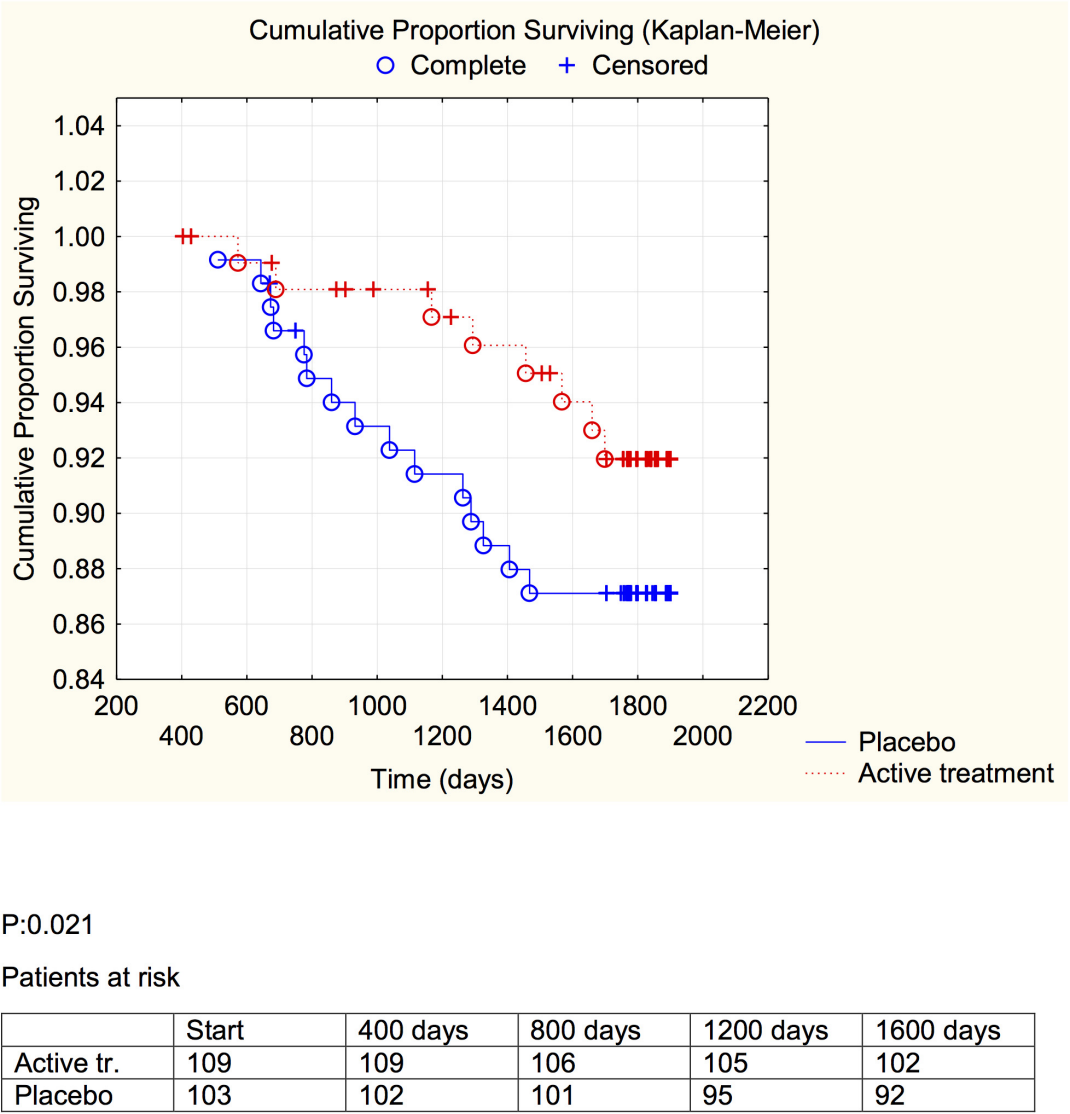
Distribution of cardiovascular mortality among those with a C-reactive protein concentration below median in those who received active supplementation of selenium and coenzyme Q10 combined versus placebo during follow-up of 5.2 years.

**Fig 5:**
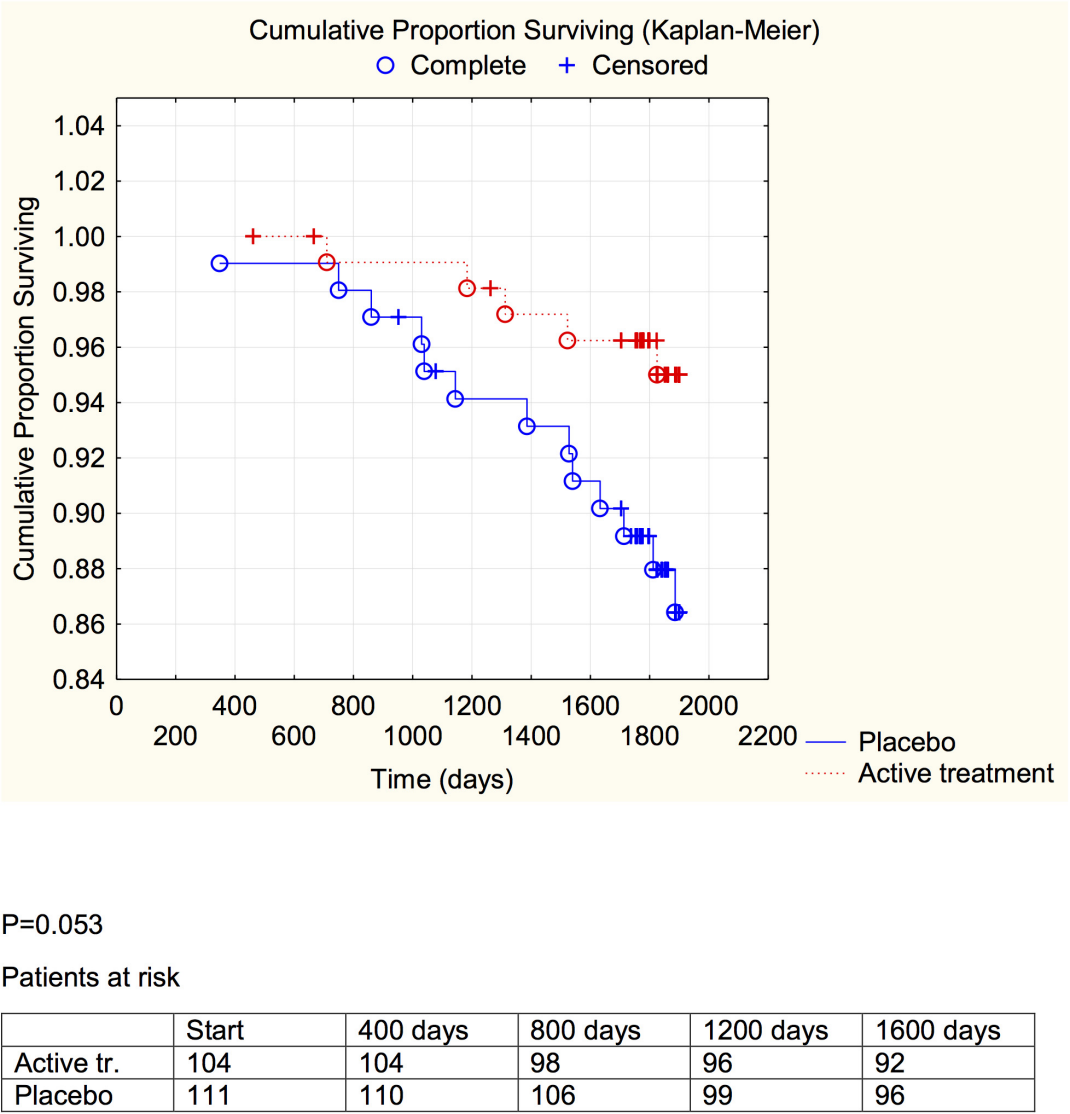
Distribution of cardiovascular mortality among those with a C-reactive protein concentration above median in those who received active supplementation of selenium and coenzyme Q10 combined versus placebo during follow-up of 5.2 years.

**Fig 6:**
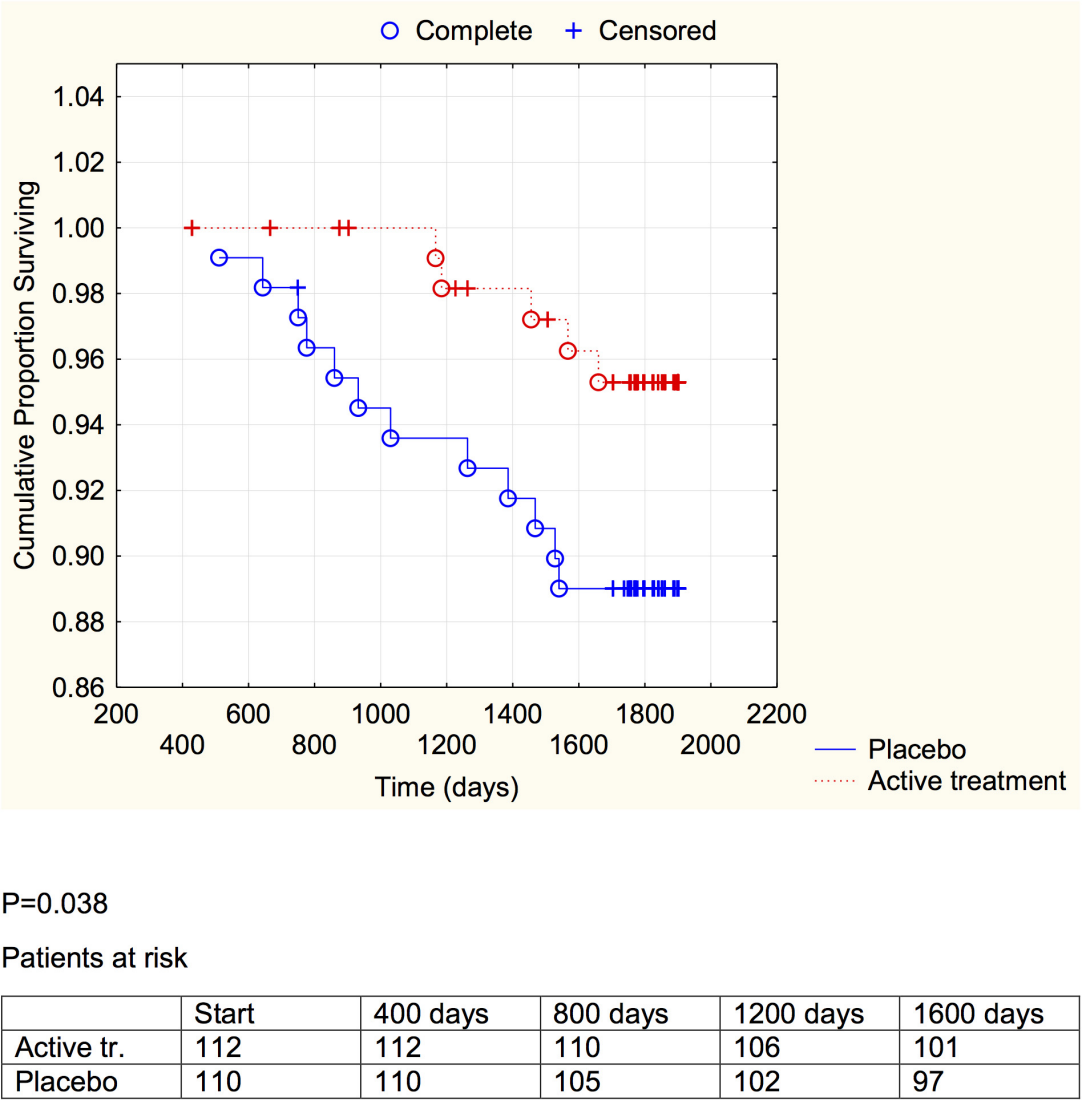
Distribution of cardiovascular mortality among those with a sP-selectin concentration below median in those who received active supplementation of selenium and coenzyme Q10 combined versus placebo during follow-up of 5.2 years.

**Fig 7:**
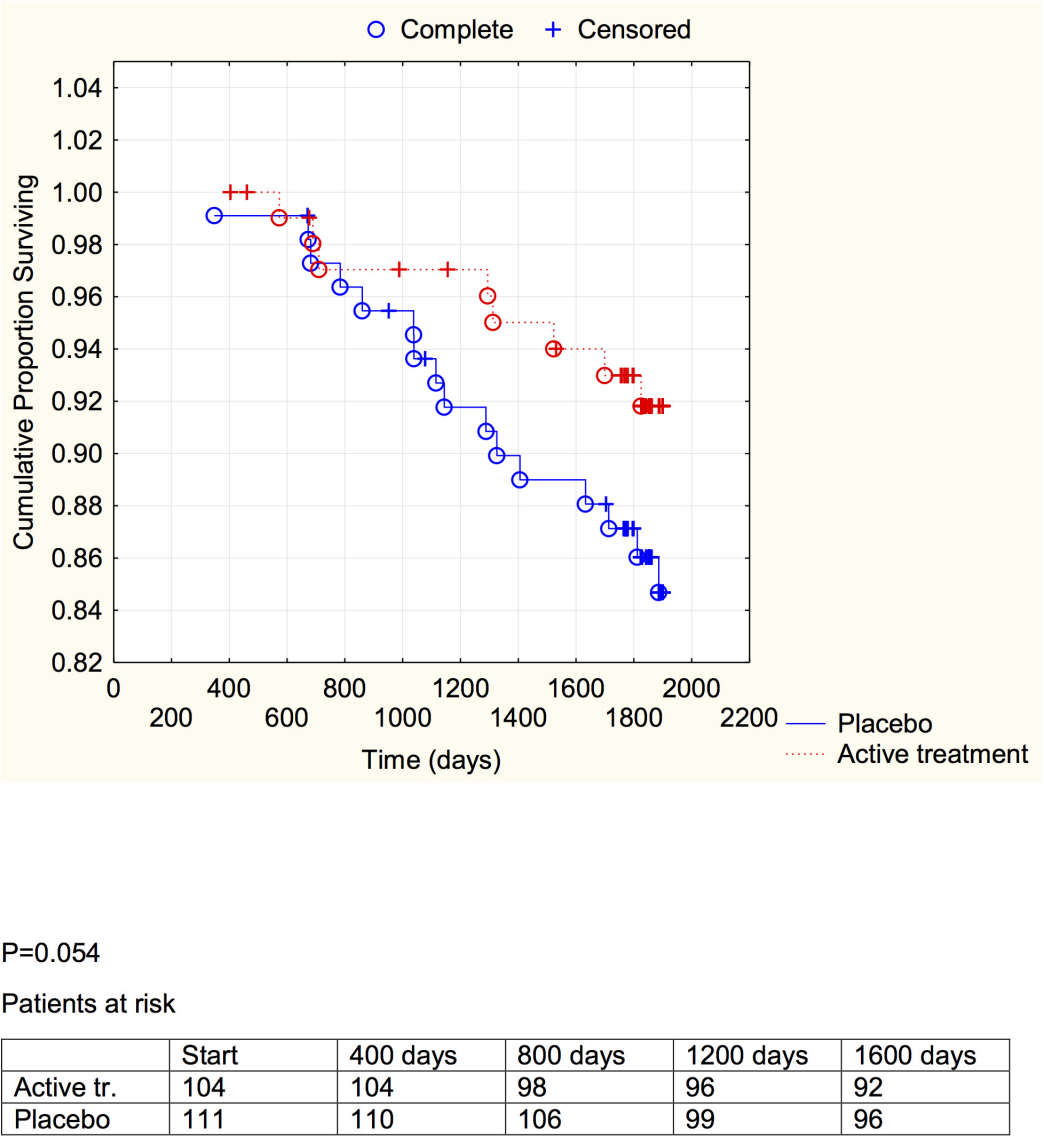
Distribution of cardiovascular mortality among those with a sP-selectin concentration above median in those who received active supplementation of selenium and coenzyme Q10 combined versus placebo followed during 5.2 years.

The extent of cardiovascular mortality in the active treatment group was about half of that in the placebo group, even though the size of the samples was small (Table 3). Using Kaplan-Meier graphs we also demonstrate the cardiovascular mortality during the study, in the active treatment and the placebo groups, evaluating the group having CRP or sP-selectin above and below median levels of the two biomarkers (Figs 4–7).

A significant difference between the active treatment and placebo groups could be demonstrated in the groups with the inflammation markers below median (CRP: *P* = 0.021; sP-selectin: *P* = 0.038), and a weaker evidence, significant at the *P*<0.1 (CRP: *P* = 0.053; sP-selectin; *P* = 0.054) was found in the groups with above median concentration of the biomarker.

## Discussion

In the present study, the dietary supplementation with selenium and coenzyme Q_10_ combined reduced the inflammatory response in elderly humans, as judged from measurements of CRP and the soluble part of sP-selectin.

We have previously reported an increase of sP-selectin in elderly community inhabitants as the participants became older [35], and this could be verified in the present study. Interestingly, the mean concentrations of sP-selectin as well as of CRP decreased during the intervention with selenium and coenzyme Q_10_ (Table 2), compared to the placebo group. We also performed evaluations using repeated measures of variance and thereby observed a significant difference between the active treatment, and the placebo groups, with lowered inflammatory biomarkers in the actively treated individuals. In the placebo group a small increase in both CRP and sP-selectin could be seen, which might reflect an age-related increase in the atherosclerosis process. However, the combined intervention appeared to protect against cardiovascular mortality irrespective of the increase in the inflammatory biomarkers (Table 3). Kaplan-Meier analyses of cardiovascular mortality were made separately for sP-selectin and CRP, each divided in two at their median value, a protective effect of active intervention with selenium and coenzyme Q10 could be revealed in both groups, although this was of marginal significance in those with the higher inflammatory response.

The mechanisms behind the effect of intervention with selenium and coenzyme Q10 combined, could be a result of several reactions:

It should be noted that the mean serum selenium concentration in the trial participants at baseline was low, viz. only about 67 μg/L [36]. The activity of the protective extracellular selenoprotein P does not plateau until a serum selenium concentration of about 120 μg/L is reached[37], and platelet GPX requires comparable selenium levels to be optimized [38]. Thus, selenoproteins that reduce oxidative stress and inflammation and thereby protect endothelial cells may operate sub-optimally in this un-supplemented elderly population, whereas supplementation may have raised and optimized platelet GPX and extracellular selenoprotein P in the majority of treated individuals.

Previous studies in comparable groups have shown that selenium supplementation decreased NF-κB activation and down-regulated the expression of inflammatory genes [39, 40].

The two selenoproteins GPX and thioredoxin reductase have been shown to protect endothelial cells from oxidants including oxidised LDL [41, 42]. In addition, selenoprotein P, which is recruited to the endothelial cells in areas of inflammation, scavenges inflammatory agents and shields endothelial membranes [43, 44]. Furthermore, another selenoprotein, selenoprotein S, plays a key role in the control of the inflammatory response [45]. Thus, a decreased inflammatory response may result from selenium supplementation alone. However, we consider it to be plausible that the observed anti-inflammatory effect results from a synergistic action of selenium and coenzyme Q_10_[11, 27]. Also, in an interventional study administering coenzyme Q10 to forty-three patients with coronary artery disease, Lee et al. reported signs of reduced oxidative stress as seen by decreased levels of malondialdehyde by 28% [46]. The correlations between CRP and sP-selectin in our groups were low or insignificant: for the placebo group the correlation was .27 (*P* = .007) and for the treatment group it was insignificant (r = .03). This indicates that the common variance was seven percent in the placebo group, and in the treatment group CRP and sP-selectin responses were independent. Accordingly, the different anti-inflammatory processes reflected by the two biomarkers were independent or differentiated in the treated group. Correspondingly, activation of platelets and inflammation form different parts of the atherothrombotic process, and the different parts may not have been synchronized in the treated group. However, our analyses suggest that intervention with selenium and coenzyme Q_10_ is effective, irrespective of the size of the inflammatory responses in the present elderly population with suboptimal selenium status.

The selenium intake among Europeans in general is low, as a result of low selenium content in the European soil, resulting in suboptimal levels for at least two of the major selenium containing enzymes, platelet glutathione peroxidase, and selenoprotein P [20]. Moreover, as the endogenous production of coenzyme Q10 declines after the age of 20, and at the age of 80 [47], only half of the coenzyme Q10 production can be found in the heart, a benefit from supplementation could be that it restores the intracellular antioxidant and anti-inflammatory potential. Thus, the effects on CRP and sPs-selectin could be a combined result of optimised selenium-containing enzymes [14, 48–50], combined with a restoration of decreased coenzyme Q_10_ levels. The positive results of our intervention in this minor cohort may be of importance for large-scale interventions on public health in Europe.

It is therefore important to stimulate further research in the area in order to expand our knowledge regarding the possibilities to interact in the process of antioxidative defence and inflammation.

## Limitations

The study has a limited size, which is why some statistical evaluations are not meaningful, which restricts the information of this report. Also, the age span of the included participants was restricted to elderly persons, which makes extrapolations into other age groups difficult. However, we argue that the report should be regarded as a hypothesis-generating study, and as such it has interesting information that could be used in further research.

## Conclusions

In this study, blood samples from more than 440 elderly community inhabitants in an intervention study using selenium and coenzyme Q_10_ combined as a dietary supplement were evaluated regarding the biomarkers C-reactive protein, and sP-selectin, which are markers for inflammation and atherosclerosis. It could be shown that in those treated with active supplementation, a significant reduction of both CRP and sP-selectin occurred compared to those receiving placebo over an intervention period of four years. The mechanism behind this effect is probably the anti-oxidative effects of both selenium and coenzyme Q10. As previously reported, reduced cardiovascular mortality has been demonstrated, which is probably associated with the decreased oxidative state in those receiving active supplementation. However, this is a small study, and further research is needed to shade light on the mechanisms.

## Supporting Information

S1 Protocol Study protocol; see Appendix. (DOCX)
