# Supplementary material for: Levels of sP-selectin and hs-CRP Decrease with Dietary Intervention with Selenium and Coenzyme Q10 Combined: A Secondary Analysis of a Randomized Clinical Trial
Source: PLoS One. 2015 Sep 16;10(9):e0137680. doi: 10.1371/journal.pone.0137680 (PMC4574282; doi:10.1371/journal.pone.0137680)
Supplement: S1 Protocol — (DOCX) [file pone.0137680.s001.docx]

**S1. Study Protocol**

**Kisel-10- a prospective randomized double-blind placebo controlled study of the results of intervention with selenium and coenzyme Q10 combined, regarding effect with emphasis on cardiovascular mortality and morbidity.**

**Background**

The amount of patients suffering from heart failure is increasing throughout the Western hemisphere. The prognosis of heart failure is poor, in many cases worse than malignant diseases.

Several studies have demonstrated that by evaluating the plasma concentration of the natriuretic peptides that are stimulated by increased wall tension of the myocardium, it is possible to estimate the risk of cardiovascular mortality.

Scientific reports have demonstrated decreased symptoms of heart failure after selenium supplementation. Selenium, which is acquired by the body through food, often exists in low levels in the body in the northern parts of Europe due to low levels of selenium in the soil. Animal studies have shown that selenium has a positive effect on the myocardium.

Ubiquinon, commonly called Q10, has a central position in many of the enzyme systems of the body. It has recently been shown that the body requires the presence of Q10 to produce selenium containing enzymes which are central in the energy handling processes of cells.

During the study all participants will be followed-up with blood samples, a new patient record, and new tablets every six months. Evaluation of health related quality of life will be performed at inclusion, after 18 months and after 48 months. The quality of life will be evaluated using the validated instrument SF-36. At the end of the study, new blood samples and new echocardiograms will be obtained.

**Aim**

To evaluate changes in primarily cardiovascular mortality and all-cause mortality, and secondary, cardiac function as evaluated with cardiac natriuretic peptides and echocardiography after dietary supplementation of selenium and Q10, or a placebo, in an elderly population during 48 months of intervention.

**Secondary objectives**

To evaluate the cost-effectiveness of selenium and Q10 combined during an intervention of 48 months. To measure the perceived health related quality of life during intervention.

Possible health related economic consequences will be evaluated using the instrument EQ-5D.

**Endpoint**

Development of cardiovascular disease, worsening of existing cardiovascular disease, and cardiovascular mortality during intervention with selenium and Q10 in comparison with placebo.

Change of concentration of natriuretic peptides during intervention with selenium and Q10.

**Secondary endpoint**

Development of all-cause mortality during intervention with selenium and Q10.

Consumption of hospital resources during intervention with selenium and Q10 in relation to placebo.

To evaluate possible mechanisms of interventions as seen in blood samples if effects could be demonstrated as described in the primary endpoint.

**Study design**

A prospective randomized double-blind placebo controlled study.

**Randomizing**

The randomising procedure will be performed using PharmaNord’s computer assisted randomization in blocks of 6.

**Study**

All participants will be offered a selenium-containing yeast preparation corresponding to 200 micrograms of selenium/day, and ubiquinone corresponding to 200 milligrams/day, or a placebo.

During the intervention period, blood samples will be drawn every six months from all participants, and the following list of substances are to be analyzed:

1. NT-proBNP
2. Thioredoxin
3. Thioredoxinreductase
4. Lipidperoxidase
5. Panel of cytokines
6. “New” biomarkers for heart failure
7. Panels of biomarkers for inflammation, oxidative stress, and ischemia.

All blood sample results will be blinded for both the investigators and the participants.

**Inclusion criteria**

Individuals living in the municipality of Kinda, aged between 70-80 years who have accepted participation in the study, and who are expected to fulfill a study period of 4 years.

**Exclusion criteria**

Recent myocardial infarction (within 4 weeks).

Planned cardio-vascular operative procedure within 4 weeks.

Hesitation concerning if the candidate can decide for him/herself whether to participate in the study or not, or if he/she understands the consequences of participation.

Serious disease that substantially reduces survival or where it is not expected that the participant can cooperate for the full 4 year period.

Other factors making participation unreasonable, such as long/complicated transport to the Primary Health Center where the project is managed, or drug/alcohol abuse.

**Preparations**

Coenzyme Q10 ( Bio-Quinon 200mg, PharmaNord, Vejle, Denmark)

Selenium (SelenoPrecise 200 microgram, PharmaNord, Vejle, Denmark)

Placebo (PharmaNord, Vejle, Denmark)

All preparations will be ingested twice a day together with food.

The preparations will be supplied by PharmaNord, Vejle, Denmark. The preparations will be stored in a locked, dry, dark room where only the personnel involved in the study will have access. A rigorous log regarding received and delivered preparations will be kept.

**Concomitant pharmacological treatment**

Participants who have been prescribed pharmacological drugs due to concomitant diseases/medical conditions are advised to continue treatment.

Participants who have been prescribed anticoagulants containing warfarin are advised to continue treatment, but it is recommended that they analyze the INR at the start of the intervention with selenium and Q10, and again after 2 weeks. Interactions between selenium/Q10 and warfarin have been discussed in literature but have not been demonstrated.

**Where is the code list?**

The code list containing information of active treatment/placebo is kept in a sealed envelope at the University Hospital, Linköping, and the codes will not be broken before the end of the study, or in the case of an emergency where information regarding the intervention is required.

**Side effects**

Serious side effects should be reported to both the National Board of Health and Welfare and PharmaNord ApS, Denmark.

**Dropouts**

Participants choosing to discontinue the study for any reason will be followed according to the intention to treat principle. All drop-outs will be registered, as well as their reason for discontinuing participation, if the participant chooses to supply this information.

Participants who take supplements of selenium or Q10 for any reason outside the study will be regarded as dropouts.

**Ethical permission**

Ethical permission shall be accepted from the Ethical Review Board before the start of the study. Oral and written “patient” information will be given to all participants, as well as a signed copy of the consent form .

**Permission from the Swedish Medical Products Agency**

Contact has been taken with the Medical Products Agency in Sweden regarding permission to use the preparations during the intervention. However, the principal investigator of the project has been orally informed by the Agency that since this was not regarded as a trial of a medication, but rather of food supplement commodities that are readily available as commercial goods, the Agency would not review the study protocol.

**Participants**

500-600 healthy individuals as well as patients treated for various diseases and already participating in the former epidemiological heart failure study in the Kinda municipality.

**Basic power calculation**

Using the assumption that the incidence of occurrences in the placebo group during a follow up of 4 years is 40%, and 28% in the intervention group, i.e. a difference in incidence between the groups of 30% and an absolute difference of 12%, 244 individuals are needed in order to obtain a statistical power of 80% with a significance level of 5%. A dropout frequency of 15% is to be expected, which is why a minimum of 560 individuals is needed in order to obtain significant differences.

**Principal investigator**

Urban Alehagen, Resident, Dept of Cardiology, University Hospital of Linköping

**Co-investigators**

Ulf Dahlström, Professor, Dept of Cardiology, University Hospital of Linköping

Anders Rosén, Professor, Dept of Cellular Biology, University of Linköping

Mikael Björnstedt, Professor, Dept of Pathology, Karolinska University Hospital, Stockholm
